# Cadaveric Simulation of Endoscopic Endonasal Procedures: Analysis of Droplet Splatter Patterns During the COVID-19 Pandemic

**DOI:** 10.1177/0194599820929274

**Published:** 2020-05-19

**Authors:** Dhruv Sharma, Kolin E. Rubel, Michael J. Ye, Taha Z. Shipchandler, Arthur W. Wu, Thomas S. Higgins, Sarah J. Burgin, Jonathan Y. Ting, Elisa A. Illing

**Affiliations:** 1 Department of Otolaryngology–Head & Neck Surgery, Indiana University, Indianapolis, Indiana, USA; 2 Department of Otolaryngology–Head & Neck Surgery, Cedars Sinai, Los Angeles, California, USA; 3 Department of Otolaryngology–Head & Neck Surgery, University of Louisville, Louisville, Kentucky, USA; 4 Rhinology, Sinus & Skull Base, Kentuckiana Ear, Nose, and Throat, Louisville, Kentucky, USA

**Keywords:** COVID-19, nasal endoscopy, sinus surgery, skull base surgery, droplet, splatter

## Abstract

**Objective:**

The primary mode of viral transmission of severe acute respiratory syndrome coronavirus 2 (SARS-CoV-2) is thought to occur through the spread of respiratory droplets. The objective of this study was to investigate droplet and splatter patterns resulting from common endoscopic endonasal procedures.

**Study Design:**

Cadaver simulation series.

**Setting:**

Dedicated surgical laboratory.

**Subjects and Methods:**

After instilling cadaver head specimens (n = 2) with fluorescein solution, endoscopic endonasal procedures were systematically performed to evaluate the quantity, size, and distance of droplets and splatter following each experimental condition.

**Results:**

There were no observable fluorescein droplets or splatter noted in the measured surgical field in any direction after nasal endoscopy, septoplasty with microdebrider-assisted turbinoplasty, cold-steel functional endoscopic sinus surgery (FESS), and all experimental conditions using an ultrasonic aspirator. Limited droplet spread was noted with microdebrider FESS (2 droplets, <1 mm in size, within 10 cm), drilling of the sphenoid rostrum with a diamond burr (8, <1 mm, 12 cm), and drilling of the frontal beak with a cutting burr (5, <1 mm, 9 cm); however, the use of concurrent suction while drilling resulted in no droplets or splatter. The control condition of external activation of the drill resulted in gross contamination (11, 2 cm, 13 cm).

**Conclusion:**

Our results indicate that there is very little droplet generation from routine rhinologic procedures. The droplet generation from drilling was mitigated with the use of concurrent suction. Extreme caution should be used to avoid activating powered instrumentation outside of the nasal cavity, which was found to cause droplet contamination.

The severe acute respiratory syndrome coronavirus 2 (SARS-CoV-2) virus is responsible for the novel coronavirus disease 2019 (COVID-19), which has become an international pandemic through expansive community transmission. The current evidence suggests that otolaryngologists are particularly at risk for acquiring the infection secondary to the nature of the profession’s close contact with the upper respiratory tract, which harbors a high viral load.^1-3^

The primary mode of viral transmission is thought to occur via the spread of respiratory droplets, which carry virus particles approximately 0.125 microns in size.^4^ These have been documented traveling distances of greater than 2 m and contaminating surfaces on which they land.^5^ In addition, there is a significant concern for airborne transmission of SARS-CoV-2 during aerosol-generating procedures (AGPs).^6^ This has led to the recommendation from the American Academy of Otolaryngology-Head and Neck Surgery to limit elective procedures involving mucosal disruption or aerosolizing sprays, which may include nasal endoscopy, functional endoscopic sinus surgery (FESS), and endonasal approaches involving the use of powered instrumentation.^7,8^

Despite these recommendations, there is a current lack of evidence quantifying the risk associated with these procedures. The purpose of this study was to investigate droplet and splatter patterns resulting from common endoscopic endonasal procedures in a cadaver-simulated series.

## Materials and Methods

### Supplies and Equipment

This study was exempt from institutional review board because it involved the use of nonliving deidentified human cadaveric tissue specimens (IRB protocol 2004100753). The experiments in the study were all conducted in a dedicated surgical laboratory on 2 fresh-frozen cadaver head specimens prepared in identical fashion and placed in a standard supine surgical position. A direct brow incision was made bilaterally across the midline and the anterior frontal table exposed.

External ports into the frontal and maxillary sinuses were created as described below. A 4-mm round cutting burr was used to perform the external trephination opening an anterior window approximately 8 to 10 mm in size into both frontal sinuses. Entry was confirmed with endoscopic visualization of the posterior table. Next, the maxillary sinus was approached with a Caldwell-Luc approach, and a similar bony window was created with a 4-mm round cutter burr with confirmation of entry with endoscopic visualization.

**Figure 1.**
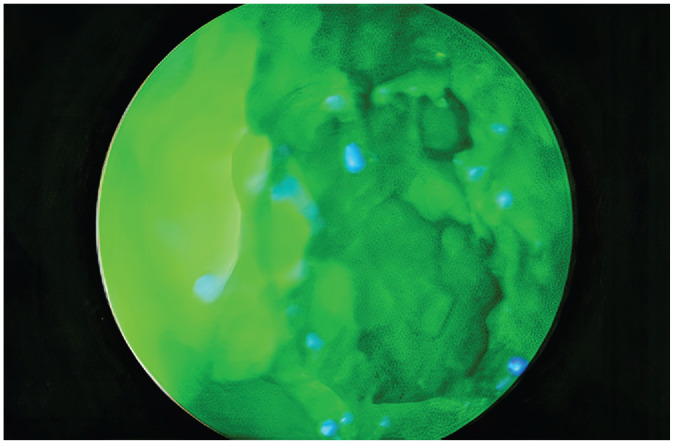
Endoscopic image of nasal cavity saturated with fluorescein.

Fluorescein solution at a concentration of 1 mg/mL was created by mixing 500 mg fluorescein 10% (100 mg/mL) AK-Fluor (fluorescein injection, USP) with 495 mL sterile saline. KERLIX gauze impregnated in Vaseline was placed transorally into the oropharynx and inferiorly through the cut tracheal edge to completely obstruct the oropharynx, hypopharynx, and larynx. The 1 mg/mL fluorescein solution was instilled using a 14-gauge angiocath through the frontal and maxillary ports. Twice the average volume of the maxillary sinus (30 mL) and frontal sinus (14 mL) was instilled into each sinus.^9^ The nasal cavity was then filled with 1 mg/mL fluorescein solution to the level of the anterior head of the inferior turbinate. After 15 minutes, a tracheal suction was used to suction out the instilled solution. The presence of residual fluorescein with adequate staining was confirmed endoscopically (**Figure 1**).

### Experimental Setup

**Figure 2.**
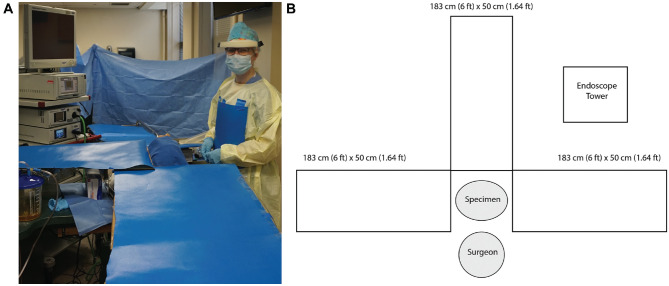
(A) Photograph of setup for the cadaveric simulation. (B) Schematic representation of experimental setup.

Each cadaver head was placed in the standard operative supine position with the right side of the head toward the right-handed surgeon. Three pieces of 183-cm (6 feet) × 50-cm (1.64 feet) nonabsorbent blue paper affixed to cardboard were placed 90 degrees from each other in the following directions: (1) superior to the head, (2) left side of the head or across from the surgeon, and (3) inferior to the head (**Figure 2A,B**). During the following experiment, a 25-cm × 25-cm piece of nonabsorbent blue paper was also affixed to the surgeon’s gown on the chest. The surgeon also wore a face-shield throughout the procedure. Immediately prior to the dissection, a tracheal suction was used to suction out any pooled fluorescein solution.

### Experiment

The senior author (E.A.I.) performed all of the experimental conditions. On the first cadaver head, the following surgical procedures were systematically performed: (1) nasal endoscopy, bilaterally; (2) septoplasty with bilateral microdebrider-assisted turbinoplasty; (3) complete left-sided FESS using cold, nonpowered instrumentation; (4) complete right-sided FESS using cold, powered suction microdebrider (Entellus Medical Shaver System SS-100; Stryker) at 5000 rpm; (5) powered drilling (Pi Drive Motor REF #5407-100-000; Stryker) of the left sphenoid face and rostrum using a 4-mm diamond burr at 75,000 rpm; (6) powered drilling of the right sphenoid face and rostrum using a 4-mm cutter burr; (7) external activation of the soiled drill; (8) powered drilling of the left frontal recess and beak using a 4-mm cutter burr; and (9) powered drilling of the right frontal recess and beak using a 4-mm diamond burr.

On the second cadaver head, the following surgical procedures were performed: (1) complete left-sided FESS using cold, powered suction microdebrider; (2) complete right-sided FESS using cold, nonpowered instrumentation; (3) powered drilling of the left sphenoid face and rostrum with size 10 Frazier suction; (4) use of a ultrasonic aspirator on the left sphenoid sinus (Ultrasonic Surgical System model UST-2001, Stryker; 100% power; 50% suction; 15 mL/min irrigation); (5) powered drilling of the right sphenoid face and rostrum using a 4-mm diamond burr with size 10 Frazier suction; (6) powered drilling of the left frontal recess and beak using a 4-mm cutter burr with size 10 Frazier suction; (7) use of the ultrasonic aspirator on the right frontal sinus; and (8) external activation of the ultrasonic aspirator. **Table 1** summarizes the procedures that were performed on the 2 cadaver heads and their duration.

**Table 1.** Droplet Splatter Results.

Procedure	Cadaver 1	Cadaver 2	Duration of procedure	Droplet or splatter contamination	Maximum contamination distance, cm	Maximum droplet size
Bilateral nasal endoscopy	x		4 minutes	No	0	0
Septoplasty and microdebrider turbinate reduction	x		12 minutes	No	0	0
Unilateral cold instrument FESS	x	x	10 minutes	No	0	0
Unilateral microdebrider-assisted FESS	x	x	10 minutes	Yes in cadaver 1 No in cadaver 2	6 in cadaver 1 0 in cadaver 2	<1 mm
4-mm round diamond burr on sphenoid bone	x		3 minutes	Yes	12	<1 mm
4-mm round cutting burr on sphenoid bone	x		3minutes	No	0	0
4-mm round burr on sphenoid bone with suction		x	3 minutes	No	0	0
Ultrasonic aspirator on sphenoid bone		x	3 minutes	No	0	0
4-mm round cutting burr on frontal beak	x		3 minutes	Yes	9	<1 mm
4-mm round diamond burr on frontal beak	x		3 minutes	No	0	0
4-mm round burr on frontal beak with suction		x	3 minutes	No	0	0
Ultrasonic aspirator on frontal beak		x	3 minutes	No	0	0
4-mm round cutting burr outside the nose	x		10 seconds	Yes	13	2 cm
Ultrasonic aspirator outside the nose	x		10 seconds	No	0	0

Abbreviation: FESS, functional endoscopic sinus surgery.

Following each of the above listed surgical procedures, the size, number, and distance of the droplets and splatter on the nonabsorbent blue paper were evaluated and measured by the following technique, and 25-cm × 25-cm transparent grid graphs were laid side-by-side until the entire length of the paper was covered. The blue paper on the surgeon’s chest was removed and laid flat, and a grid was placed on it as well. The surgeon’s face shield was removed and laid flat, and blue paper with an overlying grid was placed underneath it.

**Figure 3.**
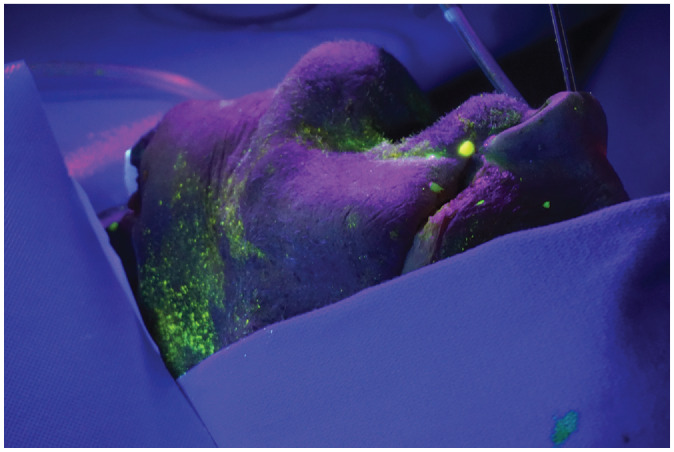
Photograph showing detection of fluorescein-stained droplets under ultraviolet light.

Since fluorescein fluoresces yellow under ultraviolet light and blue paper does not, the evaluators illuminated the paper with an ultraviolet light to visualize the droplets and splatter from each experimental condition. An example of this is depicted in **Figure 3**. Endoscopic visualization of contamination was attempted using an alternative blue filter material (Supergel Filter #74 Night Blue; Rosco Laboratories) described by Singh and Roberts.^10^ The evaluators then counted and recorded the number, size, and distance of any illuminated fluorescent spots. All measurements were performed independently by 3 evaluators (K.E.R., D.S., J.Y.T.).

## Results

No observable fluorescein droplets were noted in the measured surgical field in any direction after any of the following procedures: (1) nasal endoscopy, (2) septoplasty with microdebrider-assisted turbinoplasty, (3) FESS performed with cold instrumentation, (4) drilling of the sphenoid rostrum with a cutting burr, (5) drilling of the frontal beak with a diamond burr, (6) drilling of the sphenoid rostrum with a diamond burr with concurrent suction, (7) drilling of the frontal beak with concurrent suction, (8) ultrasonic aspirator on the left sphenoid sinus, (9) use of the ultrasonic aspirator on the right frontal sinus, and (10) external activation of the ultrasonic aspirator.

Limited droplet spread was noted under the following 4 conditions: (1) microdebrider FESS (2 droplets within 10 cm of cadaver head, all less than 1 mm in size), (2) drilling of the sphenoid rostrum with a diamond burr (8 droplets within 12 cm of cadaver head, all less than <1 mm in size), (3) drilling of the frontal beak with a cutting burr (5 droplets within 9 cm of cadaver head, <1 mm in size), and (4) control condition of the drill placed outside the nose (0.5 cm droplet on chest, 11 spots within 13 cm, largest 2 cm in size). **Table 1** summarizes which test conditions resulted in droplet or splatter contamination.

## Discussion

As the SARS-CoV-2 pandemic progresses, knowledge of how to prevent its spread is of utmost concern. With the rapid dissemination globally of the virus, anecdotal evidence from experiences in Wuhan, China, as well as historical literature from similar viral epidemics (MERS-CoV, H1N1, SARS) has guided otolaryngology protocols.^11-14^ As of the time of this publication, conflicting information persists among regulatory bodies as to degree of protection required for aerosol-generating procedures.^15,16^ While available evidence seems to suggest that spread is primarily through respiratory droplets, there is no consensus on exact transmission and little research available attempting to evaluate risk of specific otolaryngologic procedures. In otolaryngology clinical practice, many procedures involve instrumentation and examination of areas suspected to carry high viral loads: the nose, nasopharynx, and oropharynx.^17^ As practitioners begin to entertain a reverse surge into practice, knowledge of how these routine procedures generate droplets and aerosols is paramount to keeping health care providers and patients safe.

The authors found in this cadaver simulation that common endonasal procedures including nasal endoscopy, septoplasty, turbinate reduction, and FESS with cold instrumentation were not sources of droplet or splatter contamination. There were limited amounts of contamination visualized following FESS with microdebrider and powered drilling of the sphenoid rostrum and frontal beak, and all observed droplet sizes were less than 1 mm. The use of a concurrent suction while drilling resulted in no contamination. The suction was used as per routine in a normal surgical setting to suck away pooled irrigation and accumulated bone dust. The reduced pooling of irrigation may have prevented the drill from splashing and propelling droplets out of the nasal cavity. Interestingly, even in the test conditions that resulted in contamination, there was a very limited spread of droplets. In fact, the farthest contamination distance was found to be 12 cm in the drilling of the sphenoid rostrum without concurrent suction. As expected during the control condition, activating a drill outside of the nose resulted in gross contamination with a maximum contamination distance of 13 cm and larger droplet sizes. These findings suggest that the soft tissue boundaries of the nasal cavity function as a barrier in preventing splatter and droplet contamination.

In contrast to our results, Workman et al^18^ reported in a recent study positive droplet contamination after all test conditions involving the drill, which included removing bone at the sphenoid rostrum, nasal beak, and external activation each for 10 seconds. Interestingly, the single cadaver study reported no contamination with either cold or microdebrider FESS, although it is important to note that they tested each condition for 10 seconds and applied the microdebrider to the anterior and posterior septum instead of the standard FESS technique.

Rather than a contradiction of their findings, we believe our results add further context to this complex issue due to key methodological differences. All dissections in our study were performed using standard operating technique, minimizing use of powered instrumentation external to the patient or anteriorly in the nose. During the experimental conditions involving a drill, the powered instrument was only activated endonasally, used with the objective of performing key rhinological procedures in standard fashion, and was performed for 3 minutes rather than 10 seconds. Through this approach, we believe our droplet distribution simulates durations seen more frequently in the operating room environment. Running a contaminated drill externally as a control condition replicated the previously reported findings in showing gross contamination. Furthermore, the droplet contamination caused by the drill was completely mitigated by the use of concurrent suction.

In an era of heightened alertness for safe technique, we must emphasize judicious utilization of powered instrumentation, which should occur only endonasally. Moreover, we recommend the use of a concurrent suction while performing powered drilling within the sinonasal cavity and anterior skull base to prevent droplet or splatter contamination. Based on these findings, the development of suction capabilities for endoscopic endonasal drills to help mitigate the risks of droplet contamination or automatic off switches when drills or debriders are moved outside of the patient may be important. In reviewing the literature, we also believe it is an important and new finding that the activation of the ultrasonic aspirator both endonasally and externally resulted in no observable droplet or splatter contamination.

Several limitations to this cadaveric study merit discussion. First, there was no assessment of forced aerosolization (such as sneezing) in the experimental model. However, it is still vital to understand the quantity, quality, and range of droplet and splatter contamination involved during these common procedures, considering respiratory droplets are considered the primary mode of transmission of SARS-CoV-2. Another limitation is that only droplets and splatter visible to the human eye were measured. Endoscopic visualization and measurement of contamination using a blue light filter was attempted, but unfortunately, the authors found it less sensitive secondary to a glare effect. Moreover, instead of a complete 360-degree assessment, the design model allowed for measurements only in the cardinal directions surrounding the specimen.

## Conclusion

Due to the high intranasal viral loads found in infections such as SARS-CoV-2, there has been much concern over the potential for transmission associated with endonasal procedures. Our results indicate that there is very little droplet generation from key rhinologic procedures such as functional endoscopic sinus surgery and transsphenoidal pituitary approaches. This droplet generation was completely mitigated with the use of concurrent suction in the anterior nasal cavity. However, extreme caution should be used to avoid activating powered instrumentation outside of the nasal cavity, as this was found to cause droplet contamination. While these findings are encouraging, further study is warranted to determine the safety of these cases in the current environment.
